# Modelling the Evolution of Pore Structure during the Disintegration of Pharmaceutical Tablets

**DOI:** 10.3390/pharmaceutics15020489

**Published:** 2023-02-01

**Authors:** Mithushan Soundaranathan, Mohammed Al-Sharabi, Thomas Sweijen, Prince Bawuah, J. Axel Zeitler, S. Majid Hassanizadeh, Kendal Pitt, Blair F. Johnston, Daniel Markl

**Affiliations:** 1 Strathclyde Institute of Pharmacy and Biomedical Sciences, University of Strathclyde, Glasgow G4 0RE, UK; 2 Centre for Continuous Manufacturing and Advanced Crystallisation (CMAC), University of Strathclyde, Glasgow G1 1RD, UK; 3 Department of Chemical Engineering and Biotechnology, University of Cambridge, Cambridge CB3 0AS, UK; 4 Environmental Hydrogeology Group, Department of Earth Sciences, Utrecht University, 3584 CS Utrecht, The Netherlands; 5 CRUX Engineering BV, Pedro de Medinalaan 3c, 1086 XK Amsterdam, The Netherlands; 6 Global Supply Chain, GlaxoSmithKline, Ware SG12 0DE, UK

**Keywords:** pharmaceutical, tablet disintegration, swelling, discrete element method, pore size

## Abstract

Pharmaceutical tablet disintegration is a critical process for dissolving and enabling the absorption of the drug substance into the blood stream. The tablet disintegration process consists of multiple connected and interdependent mechanisms: liquid penetration, swelling, dissolution, and break-up. One key dependence is the dynamic change of the pore space in a tablet caused by the swelling of particles while the tablet takes up liquid. This study analysed the changes in the pore structure during disintegration by coupling the discrete element method (DEM) with a single-particle swelling model and experimental liquid penetration data from terahertz-pulsed imaging (TPI). The coupled model is demonstrated and validated for pure microcrystalline cellulose (MCC) tablets across three porosities (10, 15, and 22%) and MCC with three different concentrations of croscarmellose sodium (CCS) (2, 5, and 8% *w*/*w*). The model was validated using experimental tablet swelling from TPI. The model captured the difference in the swelling behaviour of tablets with different porosities and formulations well. Both the experimental and modelling results showed that the swelling was lowest (i.e., time to reach the maximum normalised swelling capacity) for tablets with the highest CCS concentration, $c_{\mathrm{CCS}}$ = 8%. The simulations revealed that this was caused by the closure of the pores in both the wetted volume and dry volume of the tablet. The closure of the pores hinders the liquid from accessing other particles and slows down the overall swelling process. This study provides new insights into the changes in the pore space during disintegration, which is crucial to better understand the impact of porosity and formulations on the performance of tablets.

## 1. Introduction

About 90% of orally consumed pharmaceutical products [1] are administered in the form of a tablet to deliver the active pharmaceutical ingredient (API) [2]. The most-common tablets are manufactured by compacting a formulated powder blend that is composed of one drug substance and a number of different excipients [3]. The physical and mechanical properties of tablets, such as porosity and mechanical strength, are significantly affected by the selected formulation and the process conditions used to make the tablet compact [4]. The compaction of the powder blend is of critical importance for the particle–particle interaction, as the particles experience intensive deformation during compaction and start to bond together through van der Waals forces, mechanical interlocking, and the formation of solid bridges [5].

The physical properties and mechanical strength of the tablet control its disintegration behaviour, which is critical for dissolving and enabling the absorption of the drug substance into the blood stream. The tablet disintegration process consists of multiple connected and interdependent mechanisms: liquid penetration, swelling, dissolution of excipients and drug, and break-up. The importance of each process depends on the formulation and process conditions used. One of the most-critical processes is the liquid penetration through the porous tablet structure, which initiates the swelling of the particles in the tablet. This swelling builds up an internal stress, which causes the break up of the tablet into smaller agglomerates and primary particles [6,7]. For the tablet to disintegrate, the internal swelling stress must exceed the strength of the bonds that are formed during compaction [6]. The liquid penetration rate is strongly influenced by the tablet porosity, i.e., it generally increases with increasing porosity [8]. In many cases, liquid penetration is the controlling mechanism for tablet disintegration, i.e., the time it takes for the tablet to disintegrate highly depends on the liquid uptake. It is important to note that there is a strong interdependence between these different disintegration mechanisms, e.g., particle swelling will cause a change of the pore structure, which will directly affect the liquid penetration process [6].

During the development of a drug product, the formulation and process conditions must be selected to deliver a tablet with the desired properties in terms of its strength, content, and disintegration/dissolution performance. This typically requires a large number of experiments for every new product to explore the relationship between material attributes, process conditions, and performance behaviour in order to identify suitable and robust conditions for the final product. In the last decade, digital design approaches have been developed and deployed to reduce experimental effort and assist in the decision-making throughout the development cycle of new medicines [9].

Wilson et al. [10] developed a population model to describe the rate of break-up of a tablet into particles and their size distribution coupled with the Noyes–Whitney equation to predict the dissolution of particles. Masoodi and Pillai [11] developed a mathematical model based on Darcy’s law describing the wicking and swelling of paper by considering a dynamic change of porosity. Markl et al. [12] modified this model based on an empirical equation from Schott [13] for microcrystalline cellulose (MCC) particles to describe the tablet swelling and also the liquid penetration kinetics. Markl et al. [12] simplified the swelling of a tablet enlargement in the axial direction only to match their experimental setup. They showed that the capillary radius, $R_{c}$, decreases with increasing swelling. They assumed that the fractional increase in the volume of the wetted compacted powder was equal to the fractional increase in the volume of a single wetted particle.

Several studies demonstrated the use of discrete element modelling (DEM) to simulate the tablet compaction process and extract information on the interparticle forces and porosity and other properties affecting the tablet performance [14,15,16,17,18,19,20]. DEM is a particle-scale numerical method for modelling the bulk behaviour of granular materials. Many geomaterials such as coal, ores, soil, rocks, aggregates, pellets, tablets, and powders can be described by this method. DEM enables the investigation of the interaction of individual particles and the interparticle effects (stresses, deformation, thermal conductivity, creep). The most-essential element of a DEM model is the underlying particle contact model. The particle contact model is used to calculate the forces acting on particle–particle and particle–wall contacts. Both contact modes can be modelled by the same model. However, the material properties (e.g., coefficient of restitution, friction coefficient, etc.) for each contact type can differ in order to model dissimilar materials. The particle motion is calculated from the force a particle experiences based on these contact models [21]. Common contact models applied for tablet compaction simulation include the Luding elasto-plastic model [22], Storkers model [23], and Hertz–Mindlin theorem [24]. Recent studies from [14,25] showed that the Luding model is suitable for pharmaceutical materials.

DEM has also been used to model the disintegration process of tablets with the ultimate goal of predicting the drug release [26,27]. Kalný et al. [27] simulated the disintegration and dissolution of a two-component tablet with ibuprofen as the API and croscarmellose sodium (CCS) as a disintegrant. They assumed that only the CCS particles contribute to the total swelling and the swelling was only occurring in the axial direction. They simplified the swelling by assuming that all particles swell simultaneously and at a constant rate, i.e., the liquid penetration behaviour was not considered. Reference [26] developed a model for simulating the swelling and dissolution process of a polymer tablet by incorporating the Fickian diffusion of water into a particle in their DEM model. The particle was assumed to be cylindrical with swelling only occurring in the radial direction. Schtt et al. [28] developed a model to simulate tablet disintegration in the human ascending colon using a discrete multiphysics approach coupled with a smoothed particle hydrodynamics and lattice spring model.

Studies from other fields, such as hydrogeology [29,30], have used DEM to simulate similar processes. Reference [29] and Reference [30] applied DEM to simulate the swelling of superabsorbent polymer particles (SAPs) with an integrated liquid penetration model. Reference [29] simulated the swelling of a bed of SAPs using a single-particle swelling model combined with the pore finite volume method to model the liquid flow in the compacts. They developed this model further [30], where the unsaturated flow was computed using a scheme of an implicit pressure solver and an explicit saturation update. Reference Braile et al. [31] developed a DEM model for the swelling of granular materials (MCC PH101, rice, and superabsorbent particles), and they simulated the swelling of the material using the first-order kinetics equation to model the swelling of single particles and the materials soaking in water.

In existing studies on tablet disintegration, the pore structure change during the disintegration process and the effect of the dynamically changing pore structure on the disintegration time are not fully understood. This study assessed the changes in the pore structure during disintegration by coupling DEM with a single-particle swelling model and experimental liquid penetration data. First, the compaction of the powders were simulated using DEM with the Luding contact model [22]. This delivers a 3D discrete element model of the tablet, which is then used to simulate the tablet swelling utilising a single-particle swelling model [32]. The use of the coupled model is demonstrated for pure MCC tablets with three porosities and MCC with three different concentrations of CCS. The model was validated against the experimental results.

## 2. Materials and Methods

### 2.1. Materials

The materials analysed in this study included MCC PH101 (Avicel PH101, Roquette, Lestrem, France) and the disintegrant croscarmellose sodium (Ac-Di-Sol, CCS, SDW-802, FMC International, Philadelphia, PA, USA). MCC, in particular grade PH101, was selected as a model compound as it is one of the most commonly used excipients in the pharmaceutical industry. The values of particles’ properties are given in Table 1 and Table 2. The particle size and sphericity were measured by QICPIC (Sympatec GmbH, Clausthal-Zellerfeld, Germany). The true density of the material was measured by a helium pycnometer (MicroUltrapyc 1200, Quantachrome instrument, Graz, Austria).

### 2.2. Experimental

#### 2.2.1. Tablet Compaction

These tablet were prepared via direct compression using a compaction simulator (HB50, Huxley-Bertram Engineering, Cambridge, UK). The samples had a diameter of 10 mm and a thickness around 2 mm. The diameter and thickness were kept constant, while the filling weight of the powder material was adjusted to vary the tablet porosity. The powder was filled manually into the die of the compaction simulator to achieve precise powder filling. The compaction process was performed using a sinusoidal compaction profile, with an average speed of 0.026 m/s. The formulations of the tablets, the compression pressure, and the porosity values are given in Table 3.

#### 2.2.2. Liquid Penetration and Tablet Swelling

**Figure 1:**
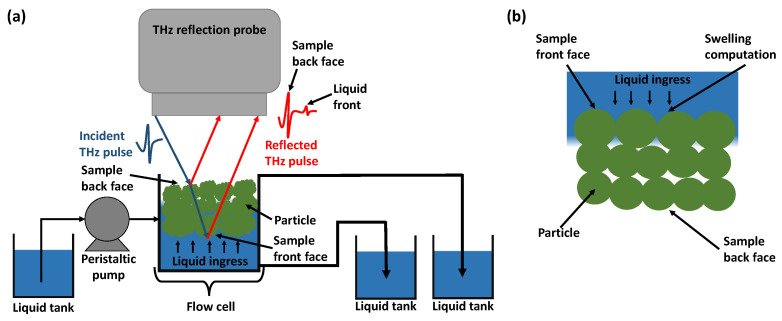
A schematic of the (**a**) experimental and (**b**) DEM modelling domain.

The liquid penetration and tablet swelling were measured using a commercial terahertz-pulsed imaging system (TPI, TeraPulse 4000, Teraview Ltd., Cambridge, UK) in combination with a bespoke flow cell [33]. The TPI was set up with a fibre-based reflection probe equipped with an 18 mm focal-length silicon lens. The probe head was on a linear scale for the ease of spatial adjustment. The beam resulting from the THz optics had a beam waist of around 1 mm at the focus with an incident angle of 13°. The TPI setup with the flow cell (Figure 1a) measures the change of the back face of the tablet, which reflects the swelling of both the back face and the front face, where the liquid uptake occurs. Since the flow cell was not included in the DEM simulation (Figure 1b), only the front face swelling was recorded in the simulation. More details about the flow cell and its design can be foundin [33].

### 2.3. Modelling

**Figure 2:**
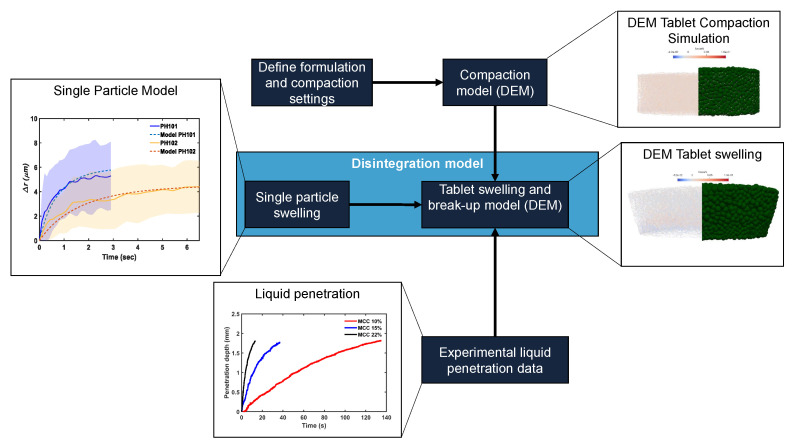
Work flow of the tablet swelling and break-up model. A single-particle model, DEM tablet compaction model, and experimental liquid penetration data are combined to model the swelling and break-up process.

Figure 2 depicts a diagram summarising the integration of the models and experimental data to simulate tablet swelling and break-up. The tablet swelling and break-up model consists of three main parts: (1) tablet compaction model in DEM, (2) disintegration model in DEM with a single-particle swelling model [32], and (3) experimental liquid penetration data. Both compaction and disintegration models were implemented in the open-source DEM software Yade-DEM [34].

#### 2.3.1. Tablet Compaction

The compaction process was simulated by compressing the particles, which were assumed to be spherical under gravity, to a loose, random packing in a cylindrical compression die with a height of 3 mm and a radius of 1 mm. To decrease the computational cost, the simulated tablet was scaled down to a diameter of 2 mm and a thickness of 0.8–1 mm while using the experimental particle size distribution (Table 2) in the DEM. The concentration of each material, the compression force, and the punch speed were set according to the experimental setup described in Section 2.2.1 and Table 3. The number of particles simulated for each formulation is given in Table 4. The compression force and porosity were recorded during the simulation.

The Luding elasto-plastic contact model [22] was applied for particle–particle and particle–wall interactions; the model is given in Equations (1)–(3). The force in the normal direction, $F_{n}$, is given as

(1) $$F_{n}=\left\{\begin{array}{cc} k_{1}\delta_{n} & \;\mathrm{if}\;k_{2}\left(\delta_{n}-\delta_{0}\right)\geq k_{1}\delta_{n}\\ k_{2}\left(\delta_{n}-\delta_{0}\right) & \;\mathrm{if}\;k_{1}\delta_{n}\geq k_{2}\left(\delta_{n}-\delta_{0}\right)\geq -k_{c}\delta_{n}\\ -k_{c}\delta_{n} & \;\mathrm{if}\;-k_{c}\delta_{n}\geq k_{2}\left(\delta_{n}-\delta_{0}\right). \end{array}\right.$$

with $k_{1}$ as the loading stiffness, $k_{2}$ as the plastic unloading stiffness, $k_{c}$ as the adhesion stiffness, $\delta_{n}$ as the normal overlap, and $\delta_{0}$ as the plastic contact deformation overlap. $k_{2}$ is defined as:

(2) $$k_{2}=\left\{\begin{array}{cc} k_{p} & \;\mathrm{if}\;\delta_{\max}/\delta_{\lim}\geq 1\\ k_{1}+\frac{\left(k_{p}-k_{1}\right)\delta_{\max}}{\delta_{\lim}} & \;\mathrm{if}\;\delta_{\max}/\delta_{\lim}<1. \end{array}\right.$$

where $k_{p}$ is the limit plastic unloading stiffness. $\delta_{\max}$ and $\delta_{\lim}$ (see Equation (3)) are the maximum compression overlap and the plastic limit, respectively.

(3) $$\delta_{\lim}=\frac{k_{p}}{k_{p}-k_{1}}\phi_{f}R^{*},$$

where $\phi_{f}$ is the dimensionless plasticity depth and $R^{*}$ is an equivalent radius. The values of the parameters used in this study are given in Table 5.

Reference [14] showed that the parameters $k_{1}$ (loading stiffness) and $k_{p}$ (limit plastic unloading stiffness) of the Luding elasto-plastic model and the particle density impacted the compression profile mostly and the adjustment of these three parameters could cover most of the variations of the compression profile. As [14] increased the particle size in the model compared to the experiments, they accounted for this in the model by calibrating the particle density. In this work, the particle size in the model was the same as in the experiments, and hence, the measured particle (true) density was used for the simulations.

The two unknown parameters in the model, $k_{1}$ and $k_{p}$, were determined through an optimisation procedure that minimises the error between the porosity values calculated from the DEM model and the experimental data. For the pure MCC tablets, this was performed for the medium compression pressure (15% porosity tablets) and was validated using the data from the experiments with low- and high-compression pressure. The calibration method was based on the work of [14]. As $k_{1}$ only affects the loading stage of the compaction process, the process was simulated for various different values of $k_{1}$, ranging from 500 to 20,000 N/m, simultaneously using the batch simulation mode in Yade. The root-mean-squared error (RMSE) between the experimental tablet porosity and simulated tablet porosity during loading for various $k_{1}$ values was minimised to identify the optimal value. Reference [19] highlighted that the difference between initial experimental porosity and that of a simulated DEM tablet is primarily caused by the deviation of the real particle shape from the assumed spherical shape in the DEM. Other factors affecting the initial packing of the powder such as intra-particle porosity and surface asperities were also not considered in the DEM. This difference in initial porosity caused an error in the estimation of $k_{1}$. To minimise the effect of this initial difference in the packing of the powder, the porosity in the loading process was scaled to a value between 0 (minimum observed porosity) and 1 (maximum observed porosity) and the calculated RMSE values were used to determine the optimal $k_{1}$ value. This scaling significantly improves the estimation of $k_{1}$ as this parameter primarily controls the curvature of the loading profile. $k_{p}$ was calibrated using the optimised $k_{1}$ value, and we simulated the process again at different $k_{p}$ to reach the final desired tablet porosity.

#### 2.3.2. Disintegration Model

The simulations of the swelling of the tablets modelled in Section 2.3.1 was set up to closely mimic the experimental work described. The time step (Δt) was set at $10^{-6}$ s/step to ensure simulation stability. The radius, mass, and inertia of individual particles were updated according to a single-particle swelling model (Equations (4)–(12)). Reference [29] originally derived this model to describe the swelling process of SAPs. The model assumes that the swelling of a particle is driven by the difference in the chemical potential between the particle and water (liquid medium) [36]. The single-particle swelling model is given as follows:

(4) $$\begin{array}{c} \frac{dr_{p,i}}{dt}=f_{w}\frac{D\varrho_{s}}{r_{p,i}\varrho_{w}}\left(\frac{Q^{\max}-Q_{i}^{\mathrm{abs}}}{Q_{i}^{\mathrm{abs}}}\right), \end{array}$$

(5) $$Q_{i}^{\mathrm{abs}}=\frac{m_{i}^{w}+m_{i}^{s}}{m_{i}^{s}}=\frac{r_{p,i}^{3}\rho_{w}}{r_{p,0}^{3}\varrho_{s}}-\frac{\varrho_{w}}{\varrho_{s}}+1.$$

where $r_{p,0}$ is the initial particle radius, $r_{p}$ is the particle radius at time *t*, $\varrho_{s}$ is the density of a dry particle, and $\varrho_{w}$ is the density of the liquid (deionised water in this study). *D* (μm${}^{2}$/s) is the diffusion coefficient for water molecules in the particle, which was assumed to be constant. $Q^{\max}$ is the maximum absorption ratio. The values of *D* (μm${}^{2}$/s) and $Q^{\max}$ for the various materials used in this study were taken from [32] and are given in Table 1.

This model assumes that the entire particle is exposed to the liquid, which is not valid when the particle is part of a compact tablet. In a compact tablet, particles form bonds with neighbouring particles across a contact area. This reduces the effective (available) surface area of the particle that is exposed to the absorbing liquid. This is accounted for by introducing the factor $f_{w}$ in Equation (4) that describes the fraction of the available surface ($A_{\mathrm{actual}}$) to the total particle surface area ($A_{p}=4\pi r_{p}$).

(6) $$\begin{array}{c} f_{w}=\frac{A_{\mathrm{actual}}}{A_{p}}. \end{array}$$

$A_{\mathrm{actual}}$ is defined as the particle surface area subtracted by the sum of the overlapping area with neighbouring particles:

(7) $$\begin{array}{c} A_{\mathrm{actual}}=A_{p}-\sum_{i}^{n}A_{\mathrm{cap},i}, \end{array}$$

$A_{\mathrm{cap}}$ is the surface area of the normal displaced volume between two neighbouring particles, particles *i* and *j*, and defined as a function of the particle, centroid *i*, $x_{i}$, $y_{i}$, $z_{i}$, and the coordinate of the contact point between particles *i* and *j* ($x_{j}$, $y_{j}$, $z_{j}$). *n* is the number of neighbouring particles. $A_{\mathrm{cap}}$ is given as

(8) $$\begin{array}{c} A_{\mathrm{cap}}=2\pi r_{p}h, \end{array}$$

with *h* as the height of the overlapping cap, defined as

(9) $$\begin{array}{c} h=r_{p}-\sqrt{{\left(x_{j}-x_{i}\right)}^{2}+{\left(y_{j}-y_{i}\right)}^{2}+{\left(z_{j}-z_{i}\right)}^{2}}. \end{array}$$

The position of the liquid was also updated every 100,000th time step using the experimental data. As the time instances of the experimental liquid penetration data did not match the simulation time points, a power law ($y=a\cdot t^{b}$) was fit to the experimental results from Section 2.2.2, enabling the calculation of the liquid front position in the tablet at the simulation time points. The fitting parameters for all formulations are shown in Table 6. The simulation of a single-particle swelling assumes that a particle starts to swell once the liquid reaches the particle centre; the model than considers the available wetted surface area to be $A_{\mathrm{actual}}$. The particle size change was implemented in the DEM by defining a growth factor (*f*):

(10) $$\begin{array}{c} f=\frac{r_{p}\left(t+M\Delta t\right)}{r_{p}\left(t\right)}=1+\frac{M\Delta t}{r_{p}\left(t\right)}\frac{dr_{p}}{dt}. \end{array}$$

To accelerate the simulation, the particle radius, mass, and inertia were updated only every 100,000th time step (M = 100,000). $r_{p}\left(t+M\Delta t\right)$ is the radius at time t+MΔt. Due to the absorption of the liquid by the particle, the mass (*m*) and inertia (*J*) of a particle were also updated:

(11) $$\begin{array}{cc} m(t+M\Delta t) & =m\left(t\right)\cdot f^{3}, \end{array}$$

(12) $$\begin{array}{cc} J(t+M\Delta t) & =J\left(t\right)\cdot f^{5}. \end{array}$$

**Figure 3:**
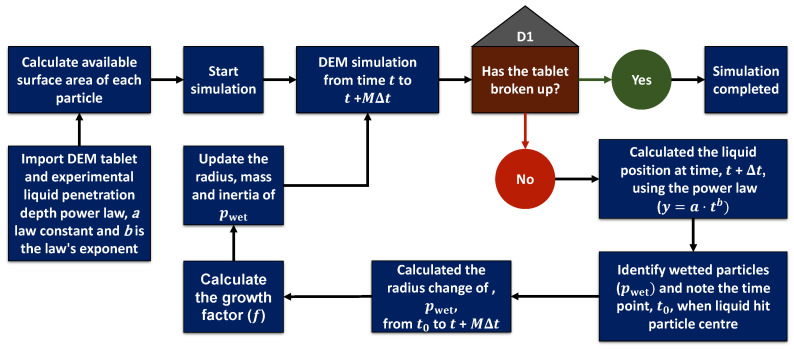
Flow chart for simulating tablet swelling using DEM incorporating a single-particle swelling model and experimental liquid penetration data.

The entire workflow to simulate the tablet swelling and break-up is presented in Figure 3.

#### 2.3.3. Pore Structure Analysis of DEM Results

The porosity was measured using the voxel porosity method [34]. This approach divides the whole volume into a dense grid of voxels at a given resolution (resolution = 200 μm) and counts the voxels that fall inside any of the particles. The porosity, ϵ, is calculated as

(13) $$\begin{array}{c} \epsilon =\frac{V-V_{v}}{V}. \end{array}$$

where *V* is volume of the tablet and $V_{v}$ is the volume of voxels that fall inside any particles.

The pore sizes were determined using the triangulation and pore finite volume method described in [37] and subsequently [29]. First, a triangulation procedure was applied to the pore space of the tablet using solid particle centres as vertices for the tetrahedra. The tetrahedron spans across four neighbouring particles and defines the pore space. This is referred to as a pore unit. The size of each pore unit in the tablet was then calculated as the radius of inscribed circle of the tetrahedron.

Cumulative porosity maps were generated to analyse the pore space spatially. This method is described in detail in [38]. In brief, PoreSpy [39] was used to generate a 3D voxel image of the tablet based on particles’ position and radius. A cuboid subsection (1400 × 1400 × 800 μm${}^{3}$) of the voxel image at the centre of the tablet was selected for this analysis. The maps were obtained by dividing the sum of the number of voxels classified as voids along each dimension (*x*, *y*, and *z*) by the total number of voxels per dimension. The generated maps depict the void fraction at each position.

## 3. Results and Discussion

### 3.1. Tablet Compaction and Parameter Calibration

**Figure 4:**
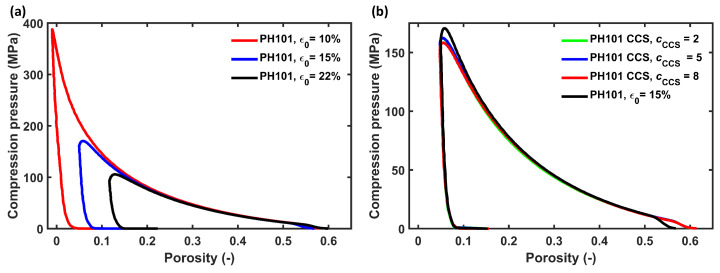
Experimental compression profile of (**a**) MCC PH101 tablet with porosities of 10%, 15%, and 22% and (**b**) MCC PH101 and CCS tablet of $c_{\mathrm{CCS}}\;=$ 2, 5, and 8 compared with MCC PH101 tablet with a porosity of 15%.

**Figure 5:**
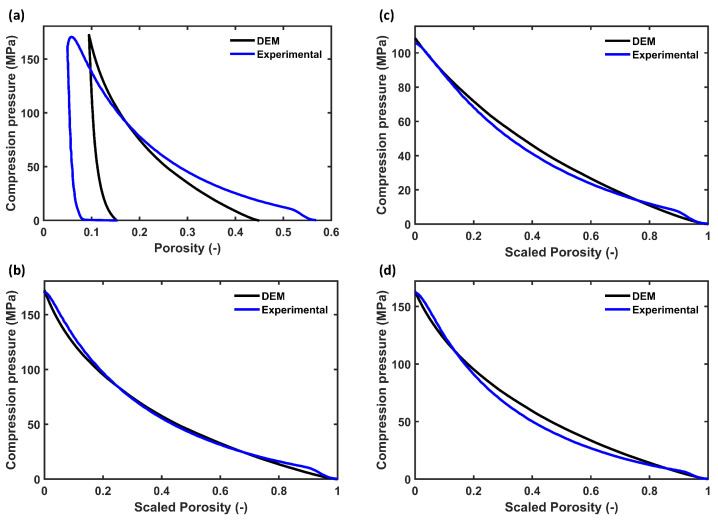
Comparison of experimental and DEM compression profiles. (**a**) Full compression profile of MCC PH101 tablet with a porosity of 15%. (**b**) Loading profile of MCC PH101 tablet with a porosity of 15% with scaled (to correct for the initial difference in porosity between experiment and DEM) porosity used for DEM parameter estimation. The validation data are shown in (**c**) for MCC PH101 $\epsilon_{0}=22\%$ and (**d**) for PH101/CCS with $c_{\mathrm{CCS}}=2$%.

The pure MCC PH101 tablet at the three different porosities and the tablet with MCC and CCS followed a very similar trend in the loading and unloading stage of the compression (Figure 4). The parameters $k_{1}$ and $k_{p}$ were thus calibrated for the MCC PH101 tablet with $\epsilon_{0}=15\%$ and validated using the profiles of other conditions. Figure 5a shows the comparison between the experimental and simulated compression profiles for an MCC PH101 tablet with $\epsilon_{0}=15\%$. As discussed in Section 2.3.2, the deviation from the measured value (ε=0.56 at t=0) and of the DEM initial porosity (ε=0.44 at t=0) influenced the parameter estimation. A scaling procedure was applied to minimise the error caused by this discrepancy. The scaled loading profiles (Figure 5b) followed similar trends, which enabled an accurate determination of $k_{1}$.

The optimisation procedure with the scaled porosity profiles of a PH101 tablet with $\epsilon_{0}=15\%$ yielded $k_{1}=10,000$ N/m, which in turn resulted in a $k_{p}=140,000$ N/m to reach the final target porosity (Table 7). The parameters were validated for MCC PH101 $\epsilon_{0}=$ 10 and 22% and MCC PH101, as well as MCC/CCS tablets with $c_{\mathrm{CCS}}=$ 2, 5, and 8% (Figure 5c,d). The DEM loading profiles followed the validation experiments closely, and the final porosity values obtained from the DEM simulation were in excellent agreement with the experimental values (Table 8).

### 3.2. Experimental Tablet Swelling and Liquid Penetration Data Analysis

The uni-directional liquid penetration profile was determined experimentally. As the experimental time instances did not match the simulation time instances, a power law ($y=a\cdot t^{b}$) was fit to each experimental dataset. The power law was then evaluated at the simulation time points to retrieve the liquid position. The fit power law parameters for all formulations studied are given in Table 6.

**Figure 6:**
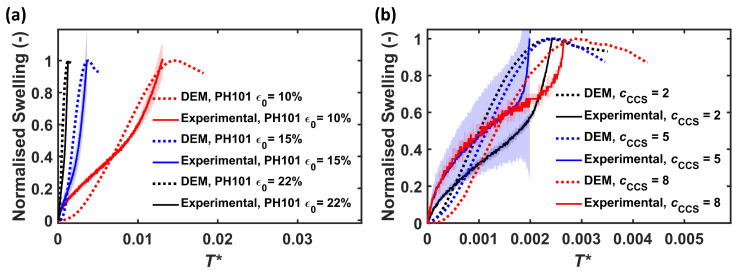
Analysis of the DEM simulation of PH101 tablets and comparison to experimental results, normalised swelling by height, as a function of normalised time, $T^{*}$. (**a**) PH101 tablets; (**b**) PH101/CCS tablets. The solid line and shaded area correspond to the average and standard deviation, respectively, of three samples (two for PH101/CCS, $c_{\mathrm{CCS}}$ = 5%).

The swelling profiles for six different conditions were simulated using the DEM with a single-particle swelling model and experimental liquid penetration depth data (Figure 6). To account for the effect of differences in thickness, $H_{0}$, between the DEM and the experimental tablet, the swelling profiles and the time were normalised. Each swelling profile was divided by its maximum. The time was normalised, $T^{*}$, using Equation (14) [29].

(14) $$T^{*}=\frac{t\cdot D}{H_{0}^{2}}.$$

As seen from Figure 6, PH101 with $\epsilon_{0}=22\%$ swelled the fastest both in the DEM and experimentally, followed by $\epsilon_{0}=15\%$ and $\epsilon_{0}=10\%$. The experimental and DEM swelling data were generally in very good agreement (Figure 6a). Both measured and DEM swelling profiles reached their maximum capacity at approximately the same time, indicating that the model captured the difference in swelling time for various tablets very well. Similar to the PH101 results, the simulated and experimental swelling profile of PH101/CCS (Figure 6b) reached the maximum swelling capacity at approximately the same time for $c_{\mathrm{CCS}}$ = 2% and PH101/CCS, $c_{\mathrm{CCS}}$ = 8%, while for $c_{\mathrm{CCS}}$ = 5%, the experimental swelling was faster.

The swelling profiles of the experiments and DEM followed different trends though. This was mainly attributed to: (1) limited consideration of the bonding types and compaction mechanisms in DEM, (2) the assumption of a spherical particle shape in the DEM, and (3) differences between the experimental flow cell and simulation setup.

Firstly, the bonds present in an MCC tablet are due to intermolecular forces such as Van der Waals force and hydrogen bonds, solid bridges, and mechanical interlocking [40]. In particular, the mechanical interlocking was not captured in the DEM simulation. Compaction phenomena such as deformation and fragmentation during compaction were also not considered in the DEM model. Reference [18] developed a method to account for the deformation during compaction. However, this was not considered at this stage as this would yield non-spherical particles in the final DEM tablet. Secondly, the single-particle swelling model was designed for spheres for simplicity and computational efficiency reasons. This is an approximation for the raw materials, as it is known that PH101 and CCS are typically non-spherical particles with a sphericity in the range of 0.66 to 0.73. The effect of the non-spherical shape on the anisotropic swelling process was discussed in [32]. Thirdly, the TPI setup with the flow cell measured the change of the back face of the tablet, which reflects the swelling on both the back face and on the front face, where it takes up the liquid [8]. The front face swelling lifts the tablet off the sample holder, causing the observed change of the back face tablet, as captured in the experimental data. As the sample holder was not included in the DEM setup, only the front face swelling was recorded in the simulation.

### 3.3. Analysis of Time-Dependent Pore Space

The swelling behaviour is primarily driven by the liquid penetration rate. The liquid penetration is strongly impacted by the change of the pore space caused by the swelling of particles. Both the pore space behind and ahead of the liquid front can change during the liquid uptake process. The data from the DEM simulations enabled the analysis of this in detail.

#### 3.3.1. Pore Space of Entire Tablet

**Figure 7:**
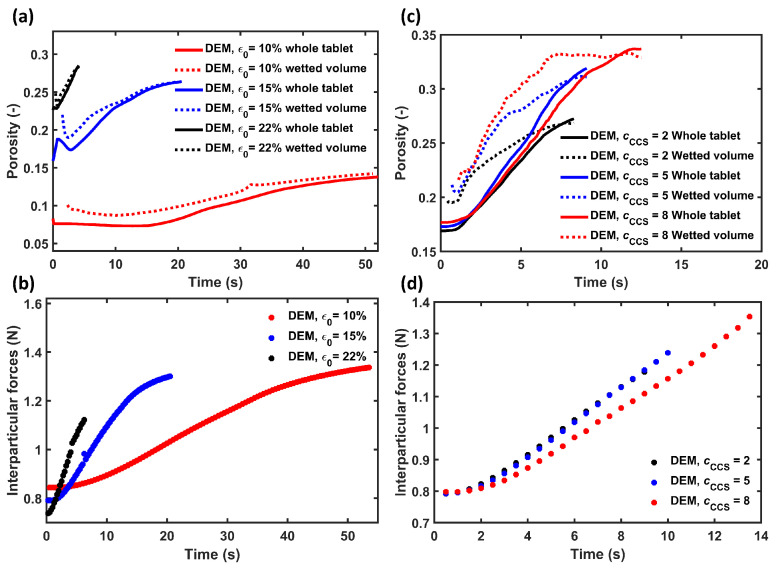
DEM simulation results. (**a**) PH101 tablets’ porosity for the whole tablet and for the wetted volume only. (**b**) Interparticle forces for PH101 tablets. (**c**) PH101/CCS tablets’ porosity analysed for the whole tablet and for the wetted volume only. (**d**) Interparticle forces for PH101/CCS tablets.

Figure 7a,c show the porosity change during the swelling of the tablets for both the overall tablet and only the wetted volume. The initial delay in the porosity measurement for the wetted volume was due to the analysed volume being too small to be accurately measured and representative. The porosity decreased initially during the swelling of the wetted volume (Figure 7a). This was observed for PH101 tablets with $\epsilon_{0}=15\%$ and $\epsilon_{0}=22\%$, indicating that the pores were closing in the initial stages of the swelling process. After the initial decrease, the porosity increased for both the wetted volume and the whole tablet. This increase was primarily attributed to the fact that the tablets were eroding, i.e., individual particles were breaking away from the tablet. For PH101/CCS (Figure 7c), the porosity of the wetted volume increased rapidly in the first three seconds. This was attributed to the fast swelling of CCS particles, resulting in a rapid increase in porosity close to the surface of the tablets. The changes in porosity were caused by particles’ movement. The changed in porosity along with the swelling of individual particles caused an increase in the tablet volume.

Unsurprisingly, the interparticle forces calculated in the DEM were strongly affected by the swelling process (Figure 7b,d). The swelling exerted stress on the particles, which caused an increase in the interparticle forces. The stress will reach a maximum, which then would lead to the disintegration of the tablet. The interparticle forces rose faster with increasing porosity (Figure 7b). For PH101/CCS (Figure 7d), the tablets with the two lower CCS concentrations had the same force profile, while the tablet with $c_{\mathrm{CCS}}\;=$ 8% experienced a slower increase of the interparticle force.

**Figure 8:**
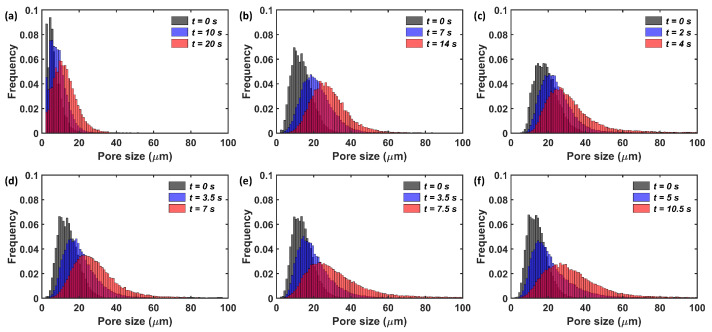
Pore size distribution at different time points during swelling of the tablet. (**a**) PH101, $\epsilon_{0}=10\%$. (**b**) PH101, $\epsilon_{0}=15\%$. (**c**) PH101, $\epsilon_{0}=22\%$. (**d**) PH101/CCS, $c_{\mathrm{CCS}}$ = 2%. (**e**) PH101/CCS, $c_{\mathrm{CCS}}$ = 5%. (**f**) PH101/CCS, $c_{\mathrm{CCS}}$ = 8%. The time points analysed: starting point (0 s), halfway point of reaching maximum swelling capacity and at the time point of maximum swelling capacity.

Figure 8 shows the pore size (pore body) distributions (PSDs) of the tablets at different time points during the swelling process. As expected, the initial average pore size was larger for tablets with a higher initial porosity, $\epsilon_{0}$, i.e., a lower compaction pressure resulted in bigger pores. The initial pore size of the PH101/CCS tablets was in the same range as the PH101 tablets. PH101/CCS with $c_{\mathrm{CCS}}=$ 2% tablets had similar PSDs as tablets with $\epsilon_{0}=15\%$, as both had approximately the same initial porosity, and the initial PSD was primarily driven by the PH101 as the major component in the tablet. As expected for the tablet with a higher CCS content, $c_{\mathrm{CCS}}=$ 5% and 8%, the number of smaller pores (∼10 μm) was higher than for $c_{\mathrm{CCS}}$ = 2%. For all tablets, the pore size increased over time, and the pores opened up shortly before the break-up of the tablet. At the maximum swelling capacity, there were pores >70 μm, which were not present at the two previous time points.

**Figure 9:**
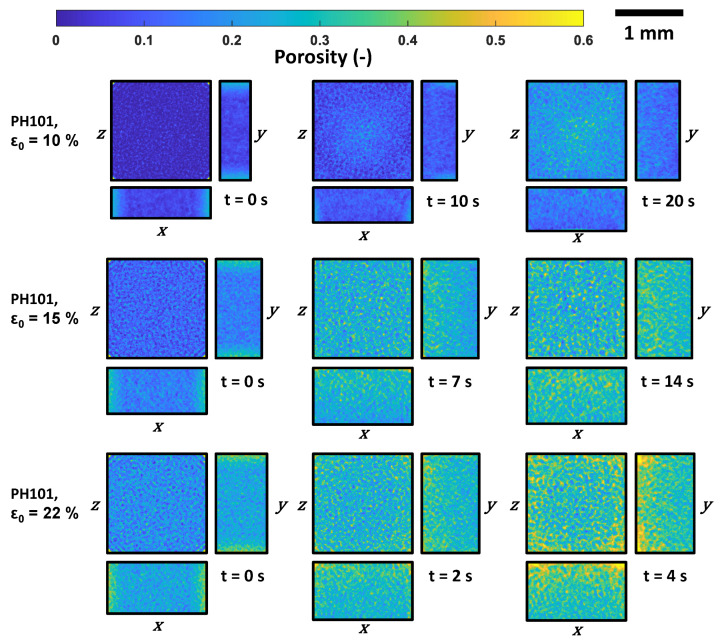
Cumulative porosity maps of the tablet at three different time points during the swelling process of PH101 tablets: Initially, halfway of reaching maximum swelling capacity and maximum swelling capacity. The cuboid subsection has a size of 1400 × 1400 × 800 pixels and was selected at the centre of the tablet for this analysis.

**Figure 10:**
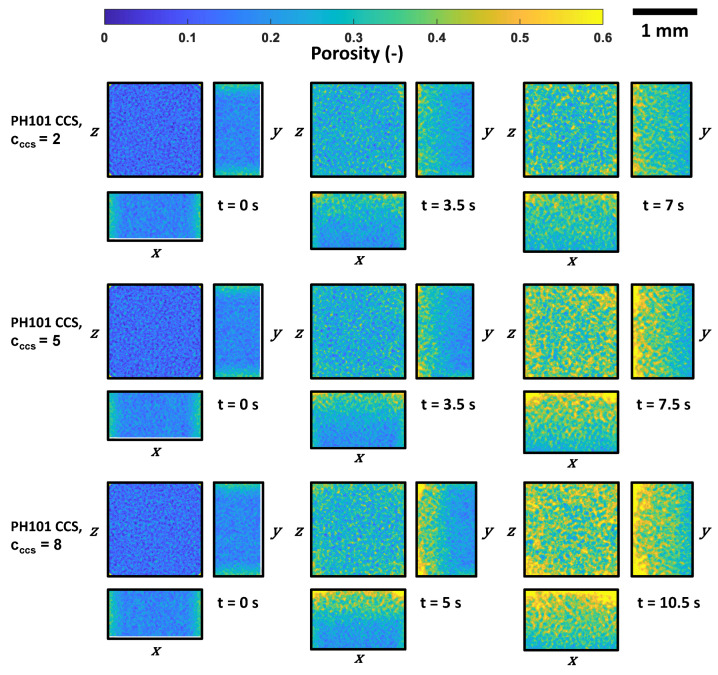
Cumulative porosity maps of the tablet at three different time points during the swelling process of PH101/CCS tablets. The cuboid subsection has a size of 1400 × 1400 × 800 pixels and was selected at the centre of the tablet for this analysis.

Cumulative porosity maps were generated to analyse the pore space spatially. This method is described in detail in [38]. In brief, PoreSpy [39] was used to generate a 3D voxel image of the tablet based on particles’ position and radius. A cuboid subsection (1400 × 1400 × 800 μm${}^{3}$) of the voxel image at the centre of the tablet was selected for this analysis. The maps were obtained by dividing the sum of the number of voxels classified as voids along each dimension (*x*, *y*, and *z*) by the total number of voxels per dimension. The generated maps depict the void fraction at each position. These maps were obtained for different time instances during the swelling process (Figure 9 and Figure 10).

As the liquid moves in one direction from the top to the bottom, resulting in the swelling of the particles from top to bottom, it is expected that the porosity will also mostly be affected along the *z* direction. However, it can be observed that the porosity changed across the entire tablet, though it had not been fully wetted.

The cumulative porosity analysis highlighted that the porosity was higher on the edges during the swelling process, which indicates that the tablet began to break up from the edges. For the PH101 tablets with $\epsilon_{0}=10\%$ and $\epsilon_{0}=15\%$, the trend in the porosity distribution was similar across the entire tablet, while for PH101, $\epsilon_{0}=22\%$, the porosity was significantly higher on the edges compared to the tablet centre.

The cumulative porosity maps for the PH101/CCS tablets were similar at the initial and the halfway times. The porosity was slightly higher at the edges for PH101/CCS, $c_{\mathrm{CCS}}$ = 8%, at the halfway point. At the final time, where the tablets reached their maximum swelling capacity, the porosity at areas close to the surface increased with increasing CCS content. This was in contrast to the overall swelling and disintegration of these tablets, where $c_{\mathrm{CCS}}$ = 8% was the tablet with the slowest swelling, i.e., the time to reach the maximum normalised swelling capacity. The disintegration of the $c_{\mathrm{CCS}}$ = 8% tablet was, therefore, not controlled by the swelling of the tablet, but the liquid uptake process, which is further discussed below. This slower liquid uptake means that a smaller number of particles were swelling for the $c_{\mathrm{CCS}}$ = 8% tablet, which in turn resulted in a slower change of the interparticle forces for this case compared to the lower CCS concentrations.

#### 3.3.2. Pore Space behind the Liquid Front

Both experiments and simulations showed a slower swelling rate for the tablet with the highest CCS content ($c_{\mathrm{CCS}}$ = 8%) (see Figure 6). These tablets also had the slowest liquid penetration rate. A similar trend was observed by [41,42], who showed that the disintegration time is prolonged with higher CCS content. As the liquid penetration rate is directly linked to the pore size, it is crucial to understand how the pore size changes in the wetted domain, as well as in the dry domain of the tablet.

**Figure 11:**
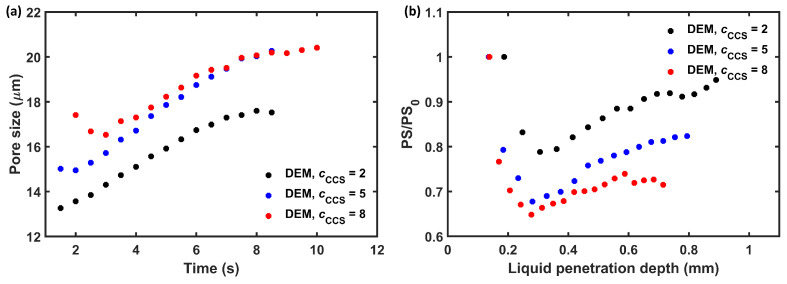
Pore size analysis of the wetted volume for PH101/CCS tablets. (**a**) The average pore size of PH101/CCS tablets at different time points during swelling. (**b**) The pore size ratio (PS/PS${}_{0}$) of the pores placed up to 0.2 mm behind the liquid front as a function of the liquid penetration depth. PS is the pore size at time t, and PS${}_{0}$ is the pore size at t=0 s.

The average pore size of the wetted domain of PH101/CCS, $c_{\mathrm{CCS}}$ = 8%, decreased during the initial stages of the swelling (Figure 11a). The pore size of PH101/CCS, $c_{\mathrm{CCS}}$ = 5%, slightly decreased initially, whereas PH101/CCS $c_{\mathrm{CCS}}$ = 2% was constantly increasing. Reference [30] explained that the reason for the decrease in the pore size (porosity) was due to the swelling of particles being much faster than that particle contacts could dissipate their potential energy. Therefore, the particle movement would be limited and the particle packing would clog (i.e., the porosity would tend to decrease). The decrease in average pore size is an indication that the pores were closing, which reduces the liquid flow. The decrease in the pore size behind the liquid front could be observed across all formulations, but it was most significant (>30% reduction in pore size) for the tablets with $c_{\mathrm{CCS}}$ = 8%. Interestingly, the pore size reduction had a nonlinear dependence on the liquid penetration depth. CCS swelled significantly faster compared to PH101, causing the closing of the pore space, which reduced the liquid flow into the tablet.

#### 3.3.3. Pore Space Ahead of Liquid Front

As the particle swelling of the material studied was omnidirectional, it can affect the dry volume of the tablet. A change in the pore space of the dry volume impacts the liquid penetration rate, which is mostly driven by the capillary action. The capillary pressure depends on the pore size at the liquid front, i.e., at the interface of the wetted and the dry volume.

**Figure 12:**
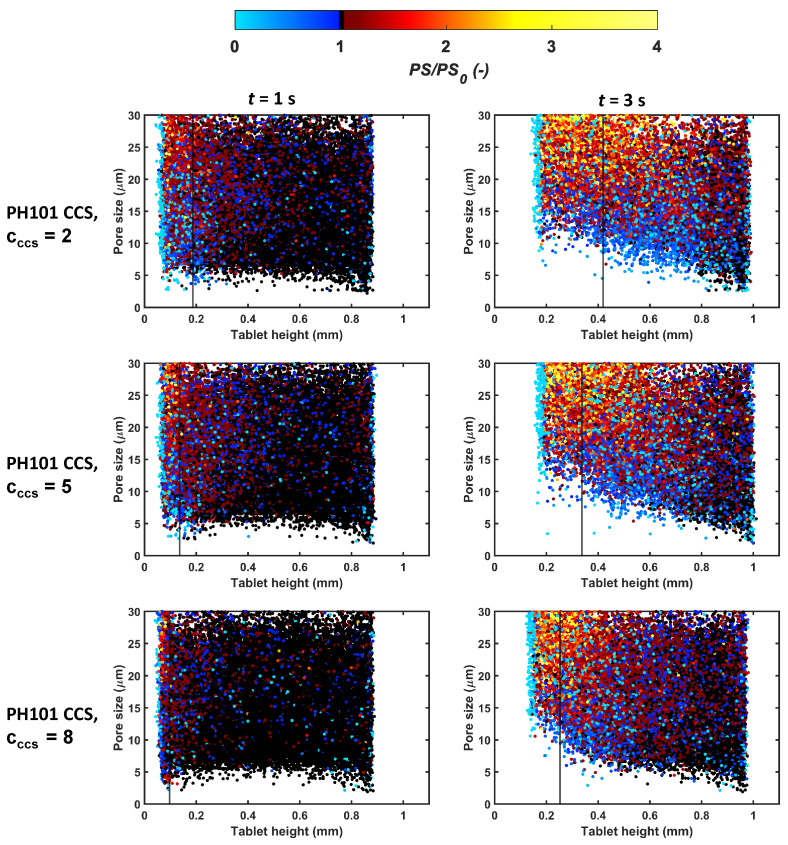
The pore size ratio (PS/PS${}_{0}$) as a function of tablet height focusing on pores <30 μm during swelling at 1 s and 3 s. The black vertical line in each plot indicates the liquid front at that particular time point. PS is the pore size at time t, and PS${}_{0}$ is the pore size at t=0 s.

The pore space of the wetted and dry volume can be analysed through the pore size ratio (PS/PS${}_{0}$) across the tablet height at different liquid front positions (Figure 12). The focus was on the smaller pores (<30 μm), as smaller pores have a greater impact on liquid flow compared to large pores.

The number of pores decreasing in size at the surface of the tablet was largest for PH101/CCS, $c_{\mathrm{CCS}}$ = 8%. At three seconds, the small pores of the $c_{\mathrm{CCS}}$ = 5% and $c_{\mathrm{CCS}}$ = 2% tablets were opening up more significantly compared to $c_{\mathrm{CCS}}$ = 8%. It can also be observed that the dry pores changed in size substantially during the swelling of the wetted volume with a decrease in the size of many small pores. Again, this phenomenon was most significant for the PH101/CCS tablet with $c_{\mathrm{CCS}}$ = 8%. The reduction in the pore size of the dry pore and wetted pores slowed down the water uptake of these tablets, which resulted in a slower swelling and ultimately delayed disintegration of the entire tablet.

## 4. Summary and Conclusions

This study demonstrated the simulation of tablet swelling by combining DEM with a single-particle swelling model and experimental liquid penetration data. The model captured the difference in the swelling behaviour of the tablets with different porosities and formulations well. For all tablets, the pore size increased over time, and the pores opened up shortly before the break-up of the tablet. Both in the experiments and DEM, the swelling was slower for tablets with the highest disintegrant concentration (PH101/CCS with $c_{\mathrm{CCS}}$ = 8%) due to the closure of the pores in both the wetted volume and dry volume. The closure of pores hinders the liquid from accessing other particles and slows down the overall swelling process.

This study provides new insights into the changes in pore space during the disintegration, which is crucial to better understand the impact of porosity and formulations on the performance of tablets. This is particularly important for formulations where the liquid uptake is performance-controlling. The interplay between the formulation, manufacturing conditions, and the dynamic change of the pore space is crucial to make informed decisions during the development of a new drug product. Having a deep understanding of the fundamental changes during the disintegration and dissolution process and its link to the formulation and process conditions can accelerate the development process and increase the robustness of the design process.

Future work will focus on the incorporation of a liquid penetration model, replacing the current need for experimental data, and a dissolution model to predict drug release as a function of time. The proposed modelling approach should also be tested and validated across a larger number of relevant materials and more complex formulations.

## Figures and Tables

**Table 1:** Values of the particle properties: size ($D_{50}$), true density ($\varrho_{s}$), shape ($S_{50}$), liquid absorption ($Q^{\max}$), and diffusion coefficient (*D*) of the two materials (obtained from [32]).

Material	$D_{50}$ (μm)	$\varrho_{s}$ (kg/m${}^{3}$)	$S_{50}$	$Q^{\max}$ (g/g)	*D* (μm${}^{2}$/s)
PH101	78 ± 1	1561 ± 3	0.73 ± 0.00	1.45 ± 0.31	396.39
CCS	54 ± 1	1403 ± 17	0.66 ± 0.01	3.16	739.75

**Table 2:** Particle size distribution of the two materials (obtained from [32]).

Material	$D_{10}$ (μm)	$D_{50}$ (μm)	$D_{90}$ (μm)
PH101	45 ± 1	78 ± 1	135 ± 1
CCS	33 ± 1	54 ± 1	90 ± 1

**Table 3:** Tablet formulations (PH101 concentration, $c_{\mathrm{PH}101}$; CCS concentration, $c_{\mathrm{CCS}}$), compaction pressure (σ), and porosity (ϵ) investigated.

Material	$c_{\mathrm{PH}101}$ (%w/w)	Material	$c_{\mathrm{CCS}}$ (%w/w)	σ (MPa)	ϵ (%)
PH101	100	-	-	387	10
PH101	100	-	-	170	15
PH101	100	-	-	105	22
PH101	98	CCS	2	162	15
PH101	95	CCS	5	162	15
PH101	92	CCS	8	158	16

**Table 4:** The number of particles simulated for each formulation.

Formulation	Number of Particles PH101	Number of Particles CCS
PH101, $\epsilon_{0}$ = 10%	11,117	-
PH101, $\epsilon_{0}$ = 15%	10,426	-
PH101, $\epsilon_{0}$ = 22%	9720	-
PH101/CCS, $c_{\mathrm{CCS}}$ = 2	10,134	720
PH101/CCS, $c_{\mathrm{CCS}}$ = 5	9699	1778
PH101/CCS, $c_{\mathrm{CCS}}$ = 8	9335	2828

**Table 5:** Summary of the DEM parameters used for all simulations. $k_{1}$ and $k_{p}$ were identified through an optimisation procedure.

Properties	Value
$k_{1}$	10,000 N/m
$k_{p}/k_{1}$	14
$k_{c}/k_{1}$	0.1 ^1^
$\phi_{f}$	0.999 ^1^
Simulation time step (Δt)	1 · 10${}^{-8}$ s
Angle of repose, PH101	0.41 rad ^2^
Angle of repose, CCS	0.69 rad ^3^

^1^ [14]. ^2^ [35]. ^3^ Measured experimentally.

**Table 6:** Parameters of the power law ($y=a\cdot t^{b}$) describing the experimental liquid penetration depth data.

Properties	*a*	*b*
PH101, $\epsilon_{0}$ = 10%	0.0535	0.7313
PH101, $\epsilon_{0}$ = 15%	0.2395	0.5470
PH101, $\epsilon_{0}$ = 22%	0.4646	0.5519
PH101/CCS, $c_{\mathrm{CCS}}$ = 2	0.1871	0.7339
PH101/CCS, $c_{\mathrm{CCS}}$ = 5	0.1359	0.8279
PH101/CCS, $c_{\mathrm{CCS}}$ = 8	0.0968	0.8739

**Table 7:** Calibrated values of loading stiffness $k_{1}$ and plastic unloading stiffness limit $k_{p}$.

Properties	Value
$k_{1}$	10,000 N/m
$k_{p}$	140,000 N/m

**Table 8:** Measured and simulated values of the tablet porosity for all conditions investigated.

Formulation	Experimental Porosity (%)	DEM Porosity (%)
PH101	9.5 ± 0.2	8.2
PH101	14.6 ± 0.2	15.3
PH101	22.0 ± 0.3	22.2
PH101/CCS, $c_{\mathrm{CCS}}$ = 2%	15.1 ± 0.2	16.7
PH101/CCS, $c_{\mathrm{CCS}}$ = 5%	15.5 ± 0.3	16.9
PH101/CCS, $c_{\mathrm{CCS}}$ = 8%	15.5 ± 0.5	17.4

## Data Availability

All data underpinning this publication are openly available from the University of Strathclyde KnowledgeBase at https://doi.org/10.15129/793370a3-78c2-4308-8c0b-beff4e997ade.

## Abbreviations

The following abbreviations are used in this manuscript:

API	Active pharmaceutical ingredient
CCS	Croscarmellose sodium
DEM	Discrete element method
MCC	Microcrystalline cellulose
PH101	Microcrystalline cellulose Avicel PH101
SAP	Superabsorbent polymer particles
THz	Terahertz
TPI	Terahertz-pulsed imaging
